# Bone Within Bone as a Calcified Subdural Hematoma

**DOI:** 10.7759/cureus.27819

**Published:** 2022-08-09

**Authors:** Majid Anwer, Anil Kumar, Anurag Kumar, Krishan K Sharma, Shreekant Bharti, Farheen Ahmed

**Affiliations:** 1 Trauma & Emergency, All India Institute Of Medical Sciences, Patna, IND; 2 Pathology, All India Institute Of Medical Sciences, Patna, IND; 3 Anesthesiology, All India Institute Of Medical Sciences, Patna, IND

**Keywords:** csdh, hemiparesis, sdh, haematoma, calcified

## Abstract

Calcified subdural hematoma (CSDH) is a very rare presentation but a known and reported entity in literature. Most of the case reports have been described in children and the elderly. Surgical treatment for CSDH is still considered controversial. We report here a case of calcified subdural hematoma in a middle-aged male that was successfully operated on. A 45-year-old male presented with complaints of right-sided weakness and seizures with a history of head trauma three years ago. Non-contrast computerized tomography (NCCT) head showed calcified subdural hematoma associated with mass effect and midline shift. A frontotemporoparietal craniotomy was done to remove the CSDH. Intra-operatively the brain was pulsating well. He was discharged on the 12th postoperative day and doing well on a follow-up visit.

## Introduction

Calcified subdural hematoma (CSDH) is a very rare presentation and is mostly found in chronic subdural hematoma. Its occurrences account for around 0.3%-2.7%. Calcification occurs in the periphery of hematoma in the chronic subdural bleed. It takes months to many years for a traumatic subdural bleed to get calcified. CSDH is characterized by the clinical features of slowly progressive neurological symptoms, including headache, decreased alertness, numbness, seizure, memory impairment, confusion, gait disturbance, and weakness [1-4]. In fact, all symptoms are almost the same as those of non-calcified CSDH. Most of the case reports have been described in children and the elderly [3-7]. In chronic cases of subdural hematoma (SDH), the incidence of calcification is much higher, and all symptomatic CSDH should be treated surgically [7]. 

## Case presentation

A 45-year-old male presented to the emergency department with complaints of right-sided weakness for one month. It was insidious in onset and gradually progressive. The patient was unable to stand and ambulate. He had a history of head trauma three years ago with complaints of seizures. He consulted with a neurosurgeon that started him on antiepileptic medication and advised surgery. However, he refused the surgery. The patient was a known case of hypertension on medication.

**Figure 1:**
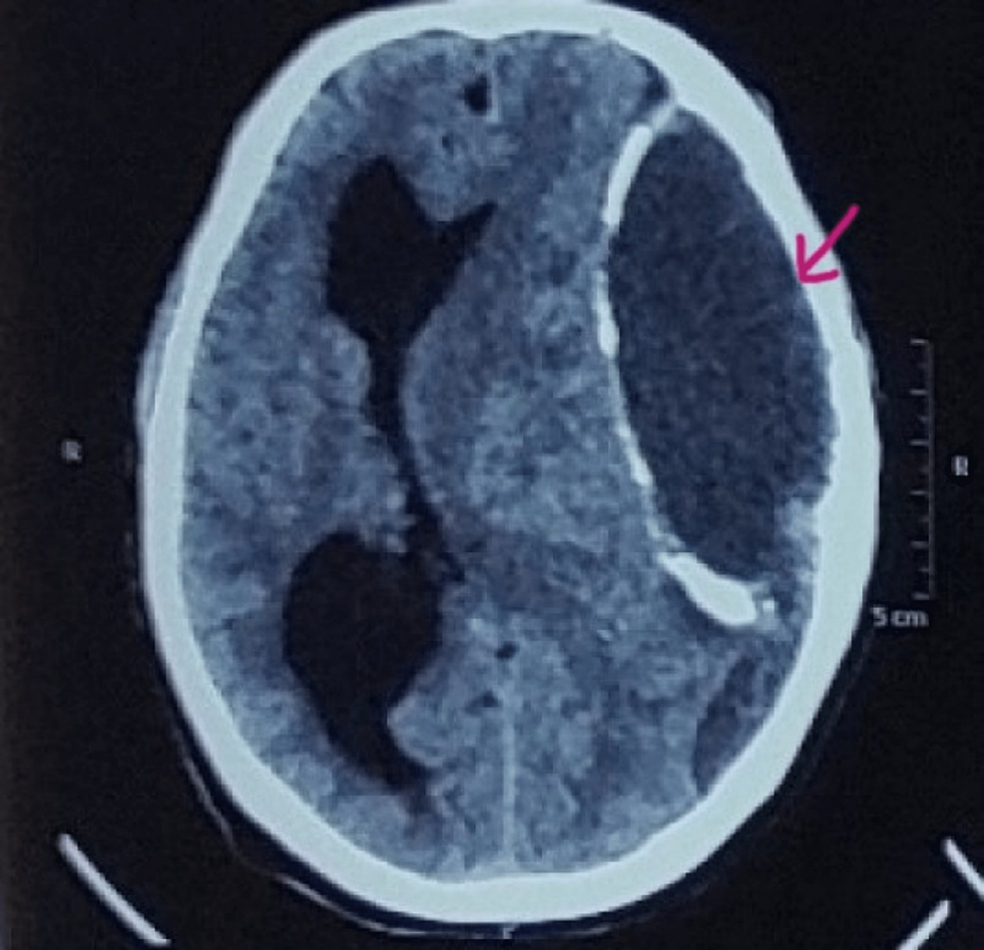
Large calcified subdural hematoma in the left cerebral hemisphere Arrow indicates large hematoma over left cerebral hemisphere with calcified inner and outer layer.

On examination, he was drowsy with a Glasgow Coma Scale (GCS) E4V4M6 and right hemiparesis with power 1/5. Non-contrast computerized tomography (NCCT) head was done, which was suggestive of subdural hematoma of the left cerebral hemisphere with calcified inner and outer layers (Figure 1) 

**Figure 2:**
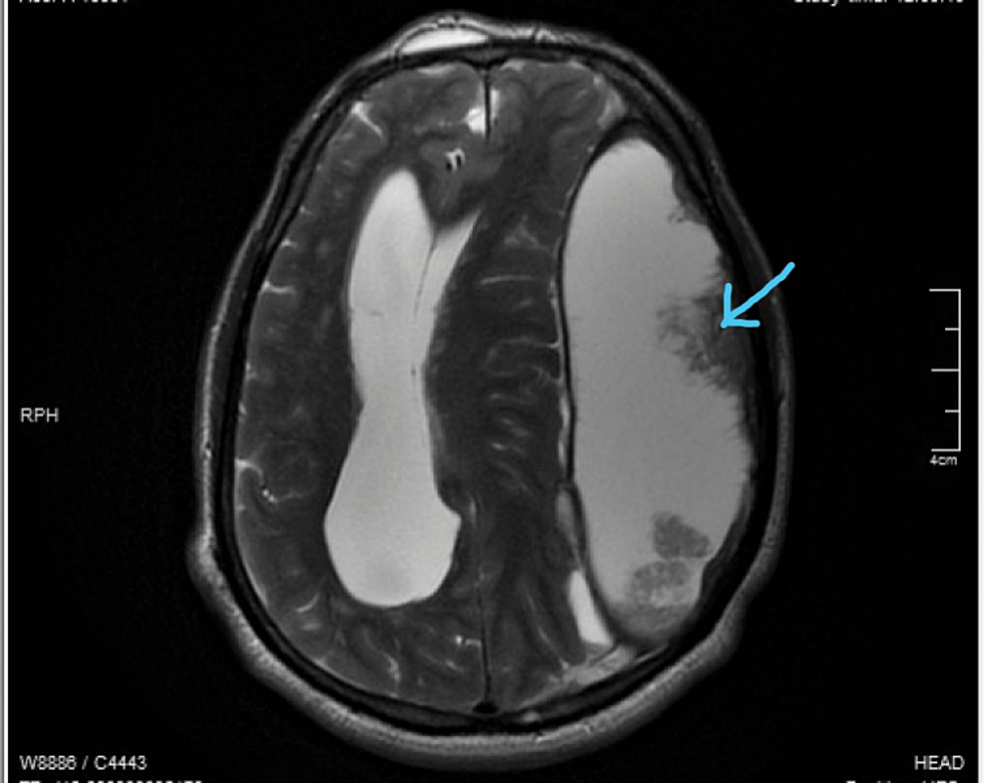
T2 weighted MRI showing a left frontotemporal hematoma with sub-acute changes Arrow indicates the sub-acute change on the left frontotemporal hematoma.

The calcified subdural hematoma was associated with mass effect and midline shift. An MRI was done, which suggested a large subdural hematoma in left frontoparietal convexity with an epidural content and significant mass effect in the form of midline shift with subfalcine and descending transtentorial herniation (Figure 2).

**Figure 3:**
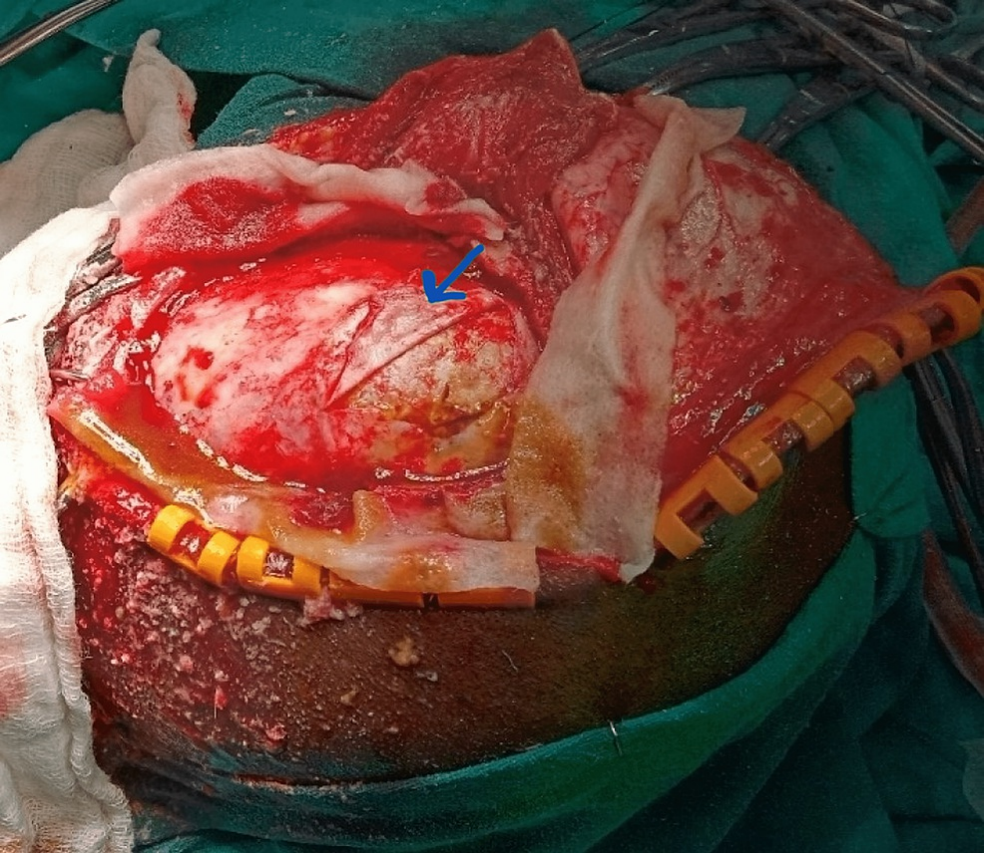
Arrow shows thinned out dura densely adherent to the outer wall of calcified hematoma

T1 and T2 signals of the hematoma suggest late sub-acute changes. There was an element of chronicity in the form of calcification of dura enclosing the collection and extensive bony remodeling of the calvaria. A lobulated lesion was noted along the superolateral margin of the epidural hematoma, which showed the post-contrast enhancement with vascular tuft within a suspicious underlying mass. A frontotemporoparietal craniotomy was done. Dura was found to be thinned out and densely adherent to the outer wall of the calcified subdural hematoma (Figure 3).

**Figure 4:**
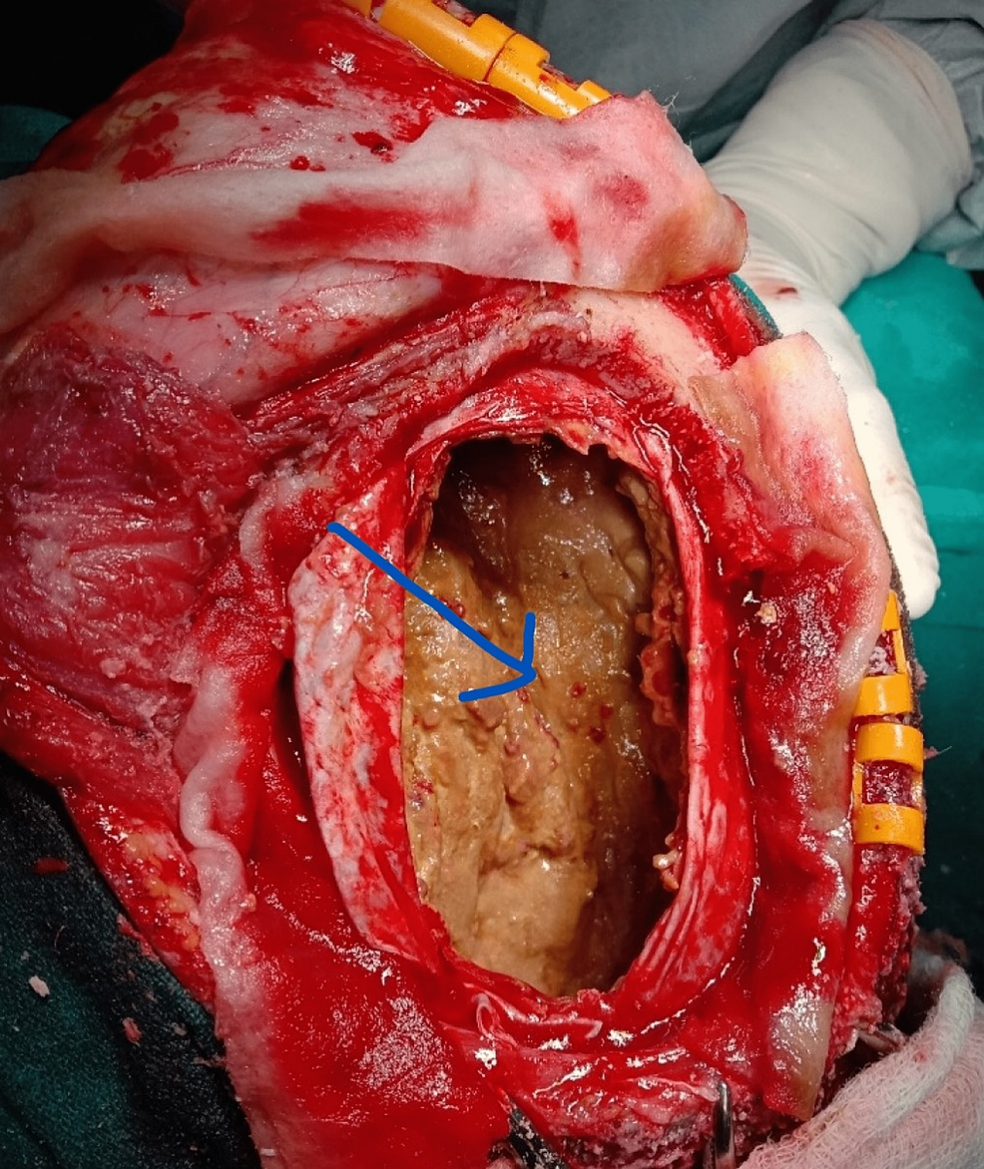
Arrow shows the inside of the subdural hematoma after removing the roof

Careful dissection was done all around, and the outer shell was removed (Figure 4).

**Figure 5:**
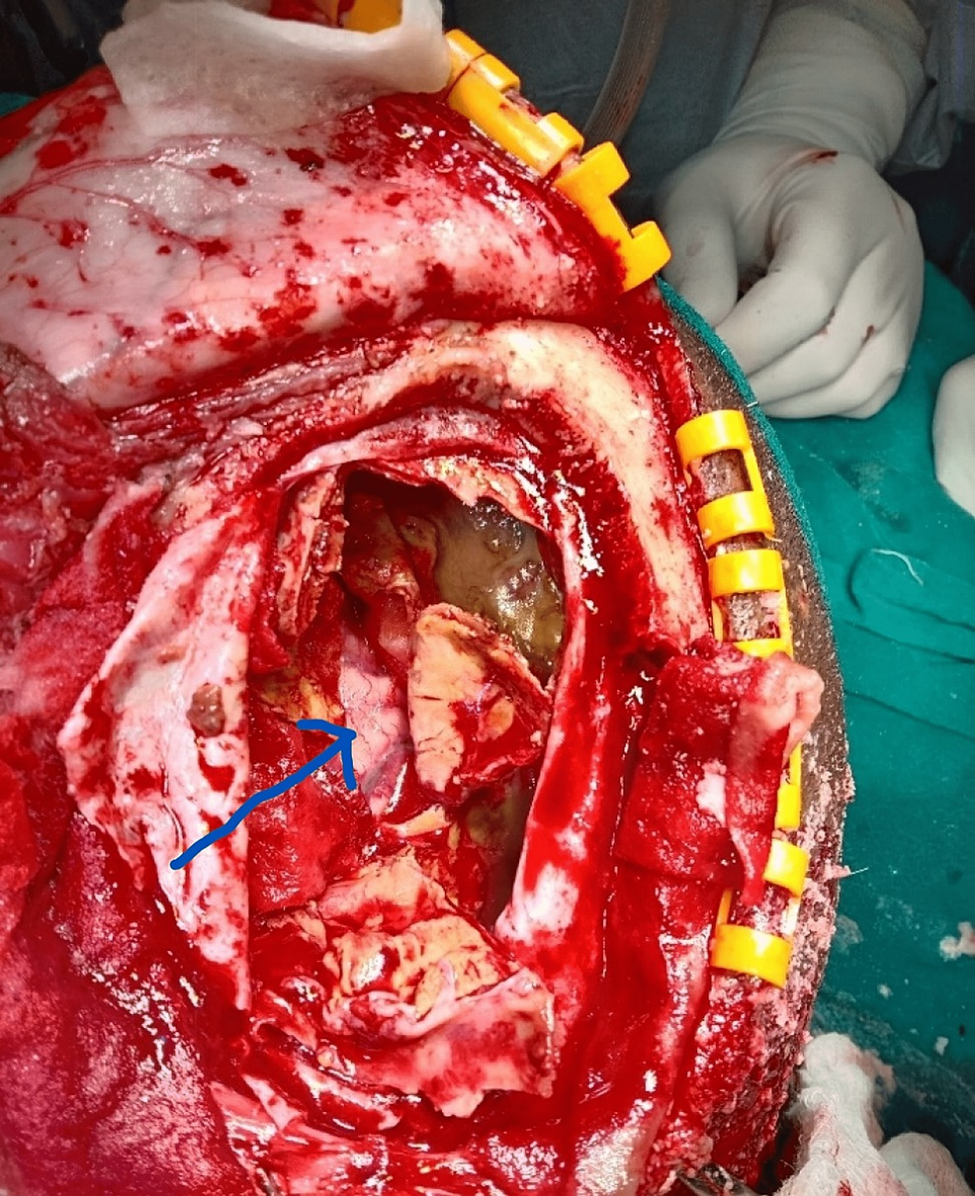
Arrow shows the posterior wall of the calcified wall being removed

Thickened, viscous, and calcified material was removed. The posterior wall was found adherent to the underlying brain parenchyma, which was carefully dissected using Penfield and bipolar cautery (Figure 5).

The medial edge was densely adherent to the superior sagittal sinus, which was left as such. Intra-operatively the brain was pulsating well.

**Figure 6:**
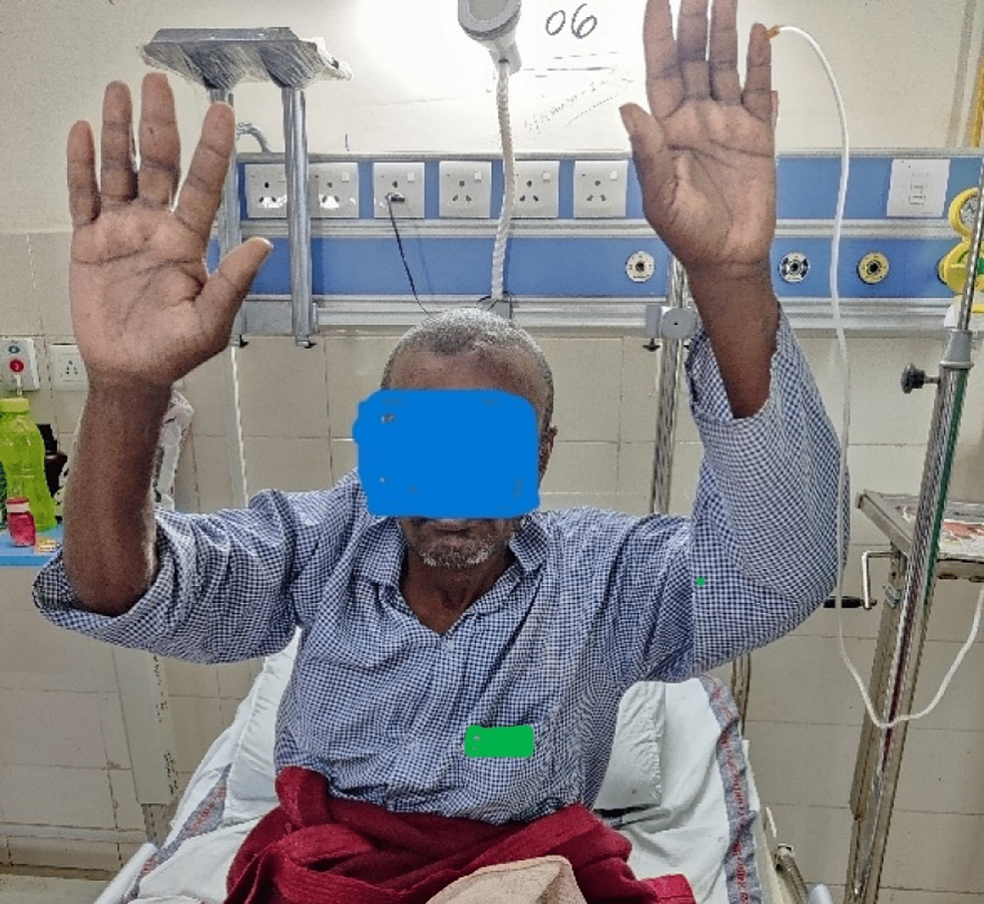
Complete resolution of weakness on the right half of the body (reproduced with permission)

During the postoperative period, the patient was extubated the next morning. He had full consciousness with GCS E4V5M6 and complete recovery of right-sided weakness (Figure 6).

The drain was removed on the third postoperative day. He developed a subgaleal collection for which percutaneous drainage was done. A postoperative CT scan showed the opening of the lateral ventricle with the resolution of mass effect and subdural collection. Biopsy of the wall of calcified hematoma was suggestive of calcified hematoma with secondary ossification (Figure 8, 9, 10). He was discharged on the 12th postoperative day. He is doing well on the fourth follow-up visit after three months of discharge from the hospital.

**Figure 7:**
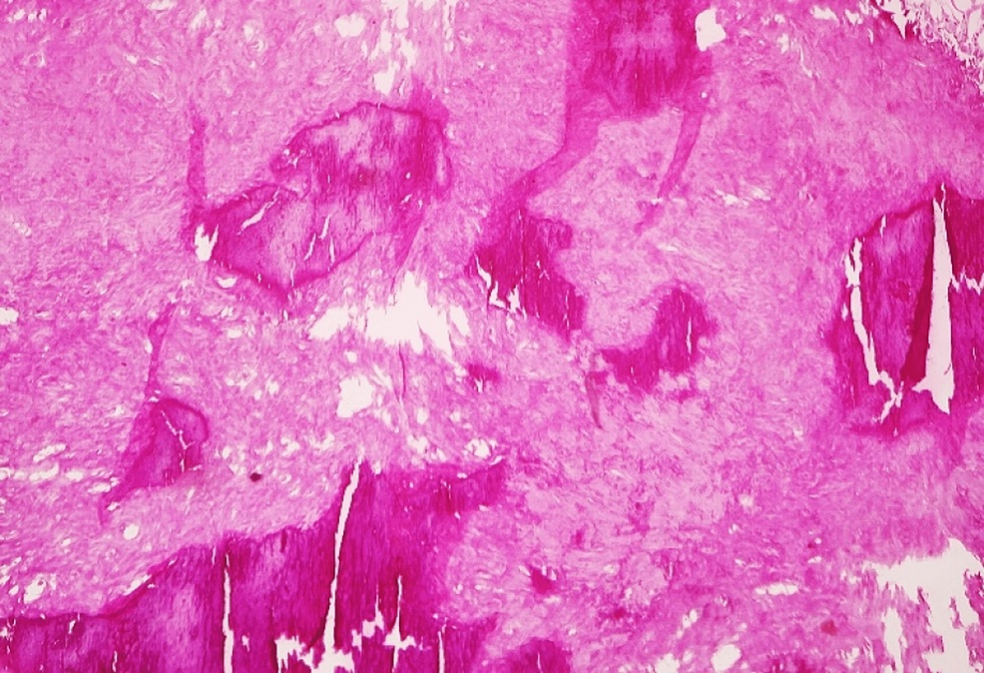
Specimen of the wall of calcified hematoma showing non-viable hemorrhagic infarct with multiple thrombosed vessels with fibrin deposition in the wall and in the extravascular tissue

**Figure 8:**
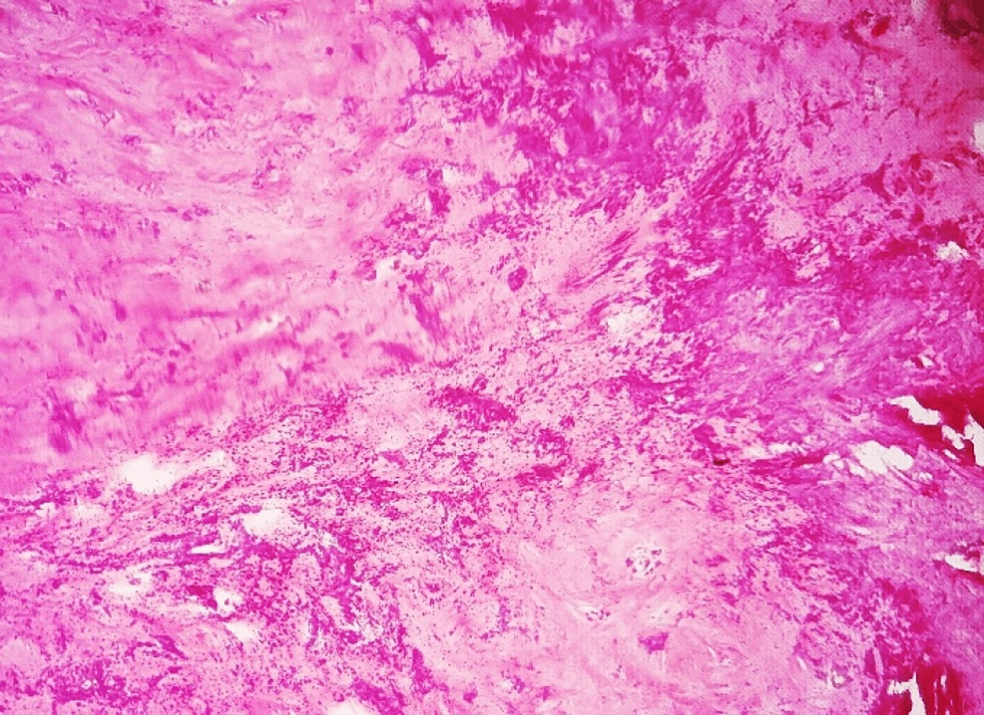
Specimen of the wall of calcified hematoma showing the organized hematoma with large areas of punctate calcification (dark purple granules); extensive hyalinization and fibrosis are present in the background

**Figure 9:**
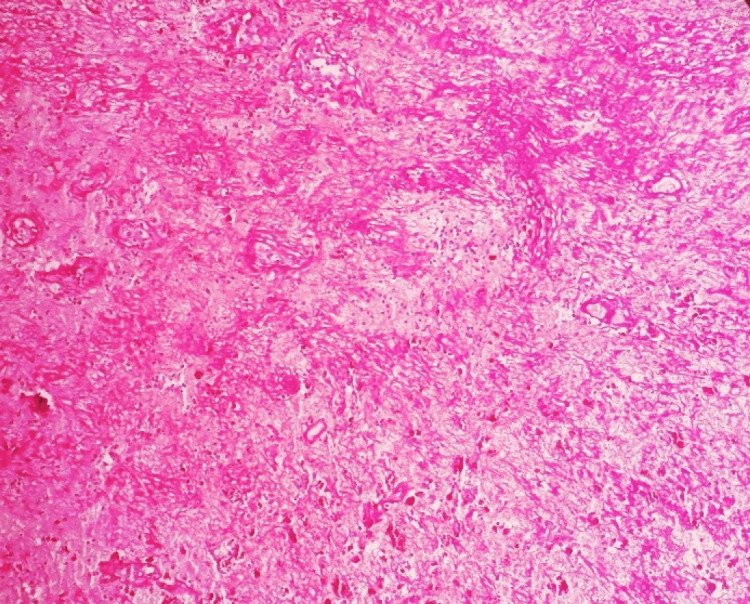
Specimen of the wall of calcified hematoma showing evidence of bizarre metaplastic bone formation was present throughout the specimen

## Discussion

Calcified subdural hematoma has been described as a case report. Around 114 case reports have been described in the literature [1-8]. As per a systematic review of 114 cases reported in the literature, it has been found that the incidence of CSDH is high in certain countries, including the United States, Japan, and Turkey, with a steady increase in recent years [8]. The reported incidence of chronic CSDH has been described to range from 0.3%-2.7% [6,7]. The incidence of chronic CSDHs is decreasing with age and is more common in children, although ages range from four months to 86 years (mean 33.7 years) [8-9]. 

CSDHs are mainly caused by head trauma in around 33.3% of reported cases, like index cases, but shunting for hydrocephalus and post-cranial surgery are other causes [8,10]. It can be unilateral or bilateral, and the area involved is cerebral convexity [6,11]. The shape can be biconvex or concavo-convex [6]. The calcification can be on the cerebral surface or on the outer surface, or bilaterally. The presentation can be varied from asymptomatic to seizure, headache, and mental retardation in children, hemiparesis, or altered sensorium [1,3,7,11]. The patient may be asymptomatic for many years, with a mean of 24.1 months [8]. In a report of two cases, there were no symptoms in children and one episode of seizure without a neurological deficit in adults [12]. The differential diagnoses include calcified epidural hematoma, calcified subdural empyema, meningioma, calcified arachnoid cyst [6,7,13], and rarely plasmacytoma or a tumor mass composed of myeloma cells [ 14,15]. One recent report says that the ossified chronic subdural hematoma should be considered a differential diagnosis when encountering an intracranial placeholder [16]. NCCT head is the primary modality of investigation and shows the characteristic calcification of the wall [7]. MRI brain is the confirmatory image for a calcified chronic subdural hematoma. Intraoperative aspiration confirmed the findings of altered fluid as there is peripheral calcification and the center of the hematoma remains liquid [9]. Calcification is a slow process and it can take from 3-12 months to many years to develop [5,6].

All symptomatic chronic subdural hematomas should be operated [7]. One reported case signified that despite the successful operation, there is a risk of fatality in contrast present case [17]. The pressure effect and the irritation goes with the removal of hematoma and causes neurological improvement [5]. Care should be taken while removing the inner wall and the medial aspect of the wall because an accidental injury to the brain or superior sagittal sinus can cause profuse bleeding.

## Conclusions

Any patients with a history of head trauma, craniotomy, and shunting should be thoroughly evaluated with imaging like CT and MRI to diagnose such rare CSDH. Once the diagnosis is confirmed, surgery is the therapy of choice in symptomatic cases, as it usually leads to postoperative improvement with the restoration of impaired neurological conditions.
